# Effects of Dietary Starch Supplementation on Muscle Metabolism and Antioxidant Capacity, Fatty Acid and Amino Acid Composition of Juvenile Yangtze Sturgeon (*Acipenser dabryanus*)

**DOI:** 10.1155/anu/7850039

**Published:** 2026-09-14

**Authors:** Tao Yan, Yunyi Xie, Xu Kuang, Tingting Wu, Bo Zhou, Qingzhi Li, Fengqi Zhao, Qiandong Li, Bin He

**Affiliations:** ^1^ Fisheries Research Institute, Sichuan Academy of Agricultural Sciences, Sichuan, 611731, China, chinawestagr.com; ^2^ Fish Resources and Environment in the Upper Reaches of the Yangtze River Observation and Research Station of Sichuan Province, Chengdu 611731, China

**Keywords:** metabolism, muscle quality, starch content, Yangtze sturgeon

## Abstract

The metabolic adaptation of endangered Yangtze sturgeon (*Acipenser dabryanus*) to dietary carbohydrates remains unclear. This study investigated how graded corn starch levels (0%, 10%, 20%, 30%) affected digestive capacity, immunity performance, nutrient metabolism and muscle quality in juveniles (216.6 ± 10.2 g) during an 8‐week trial. Digestive (lipase, amylase) and glycolytic enzymes (GK, HK, G6PDH) activities rose with starch levels, correlating with elevated glycogen storage (muscle: 8.4 mg/g; liver: 24.6 mg/g) (*p* < 0.05). Antioxidant capacity increased at 20%–30% starch (*p* < 0.05), while 30% starch reduced muscle PUFA content and increased *n* – 6/*n* – 3 ratio, elevating atherogenic (IA) and thrombogenic (IT) indices. Essential amino acid ratios and flavor‐enhancing amino acids decreased with starch inclusion, with valine as the first limiting amino acid. These findings demonstrate that 20%–30% starch could enhance metabolic and immune performance but required balancing with lipid deposition and nutritional quality considerations for sustainable Yangtze sturgeon aquaculture.

## 1. Introduction

As cost‐effective and superior carbon sources, carbohydrates incorporated into fish feed can significantly reduce the overall feed expenditure [1]. Carbohydrates are essential for various physiological processes [2, 3]. Compared to domestic animals, fish metabolize dietary carbohydrates with lower efficiency, leading to a limited carbohydrate content in commercial aquafeeds [4, 5]. The utilization of carbohydrates varies considerably among different species [6]. Optimal dietary carbohydrate levels enhance physiological condition in aquatic species by reducing catabolism of alternative nutrients (e.g., lipids and proteins), thereby improving protein utilization efficiency and reducing aquaculture production costs [7–9]. However, excessive dietary carbohydrate intake may lead to suboptimal growth performance and disrupt several physiological functions related to metabolism, immunity, and antioxidant activity [3, 10–12].

The Yangtze sturgeon is an ancient fish species mainly distributed in the Yangtze River basin of China. However, the population of Yangtze sturgeon in the natural environment has experienced a dramatic decline due to overfishing, water pollution, and habitat degradation [13, 14]. As breakthroughs in artificial reproduction technology have been achieved, research on feeding management has become a pivotal focus and a constraint in the recovery of Yangtze sturgeon resources. Feeding management, which is determined by feed composition and nutritional requirements, is directly influenced by the economic considerations of aquaculture. Research on the dietary carbohydrate levels of Yangtze sturgeon is poor. Previous research has shown that corn starch is the optimal carbohydrate source for Yangtze sturgeon and has assessed the effects of various dietary corn starch levels, ranging from 7.35% to 22.4%, which is relatively low for omnivorous fish [15]. Limited information exists regarding the physiological responses of Yangtze sturgeon to varying dietary starch levels. This study aimed to systematically evaluate the effects of different starch inclusion levels on digestive enzyme activities (trypsin, amylase, and lipase), muscle glucogen contents, muscle antioxidant capacity [superoxide dismutase (SOD), catalase (CAT), glutathione peroxidase (GSH‐PX), and malondialdehyde (MDA)], carbohydrate metabolism [glucokinase (GK), hexokinase (HK), phosphofructokinase (PFK), phospho‐pyruvate kinase (PK) and glucose‐6‐phosphate dehydrogenase (G6PDH)], and muscle nutritional quality in this endangered species.

## 2. Materials and Methods

### 2.1. Experimental Diets

Four types of semipurified isolipidic (containing 43% crude protein) and isonitrogenous (containing 10% crude lipid) diets were formulated, and they contained 0% (named T0), 10% (named T10), 20% (named T20), and 30% (named T30) corn starch, respectively (Table 1). Fish meal and casein served as protein sources. The lipid was made from soybean oil. Prior to mixing in a commercial food mixer, all the components were ground using a 200 µm mesh and were mixed with oil. The next step involved the production of 3 mm diameter pellets using a twin‐screw extruder (Zhengzhou, Henan, China).

**Table 1:** Formulation and proximate chemical composition of trial diets (% dry matter).

Ingredient (%)	Dietary starch levels (%)			
	T0	T10	T20	T30
Peru steam‐treated fish meal (670 g kg^−1^ protein)	35	35	35	35
Poultry by‐product meal	15.0	15.0	15.0	15.0
Casein (900 g kg^−1^ protein)	5.0	5.0	5.0	5.0
Gelatin	5.0	5.0	5.0	5.0
Corn starch	0.0	10.0	20.0	30.0
Soybean oil	5.0	5.0	5.0	5.0
Monocalcium phosphate	1.5	1.5	1.5	1.5
CMC‐Na	2.0	2.0	2.0	2.0
Vitamin premix^a^	0.22	0.22	0.22	0.22
Mineral premix^b^	0.25	0.25	0.25	0.25
Choline chloride	0.5	0.5	0.5	0.5
Microcrystalline celluose	30.53	20.53	10.53	0.53
Chemical composition (%)				
Crude protein	43.93	44.08	44.10	44.62
Crude lipid	10.24	10.38	10.82	10.26
Ash	10.22	10.28	10.29	10.40

^a^Mineral premix (mg kg^−1^ of diet): Na, 30; K, 50; Cu, 3.2; Fe, 150; Zn, 34; Mn, 13; I, 5.7;Se, 0.3; Co, 12.4.

^b^Vitamin premix (mg kg^−1^ of diet): VA, 20; VD_3_, 10; VE, 100; VB_1_, 5; VB_6_, 10; VB_12_, 0.02; VK_3_, 10; VB_2_, 10; inositol, 200; calcium‐D‐pantothenate, 40; niacinamide, 100; folic acid, 5; biotin, 1; ethoxyquin, 100.

### 2.2. Experimental Procedures

All fish handling and experimental procedures were approved by the Animal Care and Use Committee of the Fisheries Research Institute, Sichuan Academy of Agricultural Sciences (Approval Number 20,230,608,002A). Breeding of Juvenile Yangtze sturgeon was conducted at the Fishery Institute base of the Sichuan Academy of Agricultural Sciences, Yibin, Sichuan, China. Daily acclimation of the fish with a commercial sturgeon diet (TONGWEI Company, Sichuan, China) was performed at 9:00 a.m. and 5:00 p.m. prior to the trial. This step lasted for 2 weeks. The fish were then anesthetised using an immersion solution of 0.1 g/kg MS‐222 (Sigma, USA). Further, the fish were fasted for 24 h for weight measurement. A total of 120 fish (average weight 261.6 ± 10.2 g, mean ± standard error (SE)), which were randomly assigned to four treatment groups contained in 12 round fiber‐reinforced plastic tanks (with a diameter of 1.8 m and a water depth of 0.85 m). Each tank housed 10 fish, and during the 8‐week experiment, the fish were fed manually twice every day (9:00 a.m. and 5:00 p.m.) until they were visibly full. Feeding at a ratio of 2% of the fish body weight was conducted for 56 days. The rations were adjusted 2 weeks after fish weighing. The water flow in each tank was maintained at 250 mL/s, and a continuously aerated pool was used as the water supply. The water temperature, pH, ammonia nitrogen concentration, and dissolved oxygen levels were maintained at 19.5 ± 0.8°C, 7.3 ± 0.4, 0.15 ± 0.07 mg/L, and 6.8 ± 0.5 mg/L, respectively.

### 2.3. Sample Collection

The Yangtze sturgeons were fasted for 24 h after the completion of experiments. After the Yangtze sturgeon were anesthetised with 0.1 g/kg MS‐222, their biological data, including their length and body mass, were measured. After weighing, the Yangtze sturgeons (three fish in each tank) were dissected for muscle sampling immediately after being killed by cutting the spine. The tissue were frozen using liquid nitrogen and then stored at ‐80°C before analysis.

### 2.4. Sample Analysis

The enzyme activities of trypsin (No. F69735‐A), amylase (No. F3818‐A), lipase (No. F3817‐A), glycogen (No. F69119‐A), GK (No. F69155‐A), HK (No. F69158‐A), PFK (No. F69200‐A), PK (No. F69159‐A), G6PDH (No. F69160‐A), SOD (No. F3822‐A), CAT (No. F69001‐A), GSH‐PX (No. F69036‐A), and MDA (No. F6293‐A) were measured using commercial fish kits (Fankewei Bio, Shanghai, China) [16]. The protein concentration of the tissues was determined using BCA protein assay reagent, according to the method of Bradford [17].

For each group, dorsal muscle tissue above the vertebrae was sampled from Yangtze sturgeon for analysis of major nutritional components. Muscle nutrient composition was determined following standard procedures of the Association of Official Analytical Chemists (AOAC) [18]. Moisture content was measured by oven‐drying samples at 105°C until a constant weight was achieved. Crude protein was quantified by the Kjeldahl method for nitrogen determination, while crude ash was obtained after combustion in a muffle furnace at 550°C for 12 h. Amino acid profiles were analyzed via hydrochloric acid hydrolysis. Fatty acid composition was characterized by gas chromatography–mass spectrometry (GC/MS); individual fatty acids were identified against the NIST08 standard spectral library according to peak elution order, and the relative proportion of each major fatty acid was calculated using the area normalization method.

Amino acid score (AAS) was calculated according to the FAO/WHO‐recommended evaluation criteria to assess amino acid nutritional quality [19]. The ratio of branched‐chain amino acids to aromatic amino acids (Fischer’s ratio) was also computed for amino acid quality assessment.

(1) AAS=mg of amino acid in1g of test proteinmg of amino acid in reference pattern×100,

(2) Fischer ratio=Val+Leu+IlePhe+Tyr.

The nutritional value of fatty acids in dorsal muscle was evaluated by index of atherogenic (IA) and index of thrombogenic (IT). The degree of oxidation of polyunsaturated fatty acids (PUFAs) was evaluated by polyene index (PI).

(3) IA=C1204140160:+×C:+C:∑MUFA+∑PUFA,

(4) IT=C140160180:+C:+C:0.50.53×∑MUFA+×∑PUFAn−6+×∑PUFAn−3+∑PUFAn−6∑PUFAn−3,

(5) PI=C205226:+C:C160:.

### 2.5. Statistical Analysis

The results were suggested as mean ± standard error (M ± SE, *n* = 3). Then, the data were analyzed using SPSS software version 22.0 for Windows. Duncan’s multiple‐range post hoc test and one‐way analysis of variance (ANOVA) were conducted to determine the significant differences of the results. A significant level at 5% probability was applied in this work.

## 3. Results

### 3.1. Chemical Composition

Table 2 shows the effects of dietary starch levels on chemical composition. Findings reveal that at high starch levels, the muscle crude lipid increased significantly (*p* < 0.05), and muscle crude protein and ash under the T0 treatment were evidently larger than those in other groups (*p* < 0.05). The glucogen contents of muscle and liver showed remarkable increases with the increase in starch levels (*p* < 0.05).

**Table 2:** Chemical composition of Yangtze sturgeon fed different dietary starch levels (M ± SE).

Parameters	Dietary starch levels			
	T0	T10	T20	T30
Chemical composition				
Moisture level	77.04 ± 0.22	77.81 ± 0.44	77.76 ± 0.51	77.84 ± 0.25
Crude protein	16.80 ± 0.04^c^	16.09 ± 0.07^a^	16.16 ± 0.05^a^	16.60 ± 0.06^b^
Crude lipid	4.12 ± 0.08^a^	4.42 ± 0.17^ab^	4.46 ± 0.06^ab^	4.74 ± 0.19^b^
Crude ash	1.26 ± 0.04^b^	1.16 ± 0.02^a^	1.13 ± 0.02^a^	1.16 ± 0.03^a^
Glycogen				
Muscle glycogen (ng/g)	5.02 ± 0.11^a^	5.42 ± 0.25^b^	5.94 ± 0.13^c^	6.65 ± 0.26^d^
Hepatic glycogen (ng/g)	13.85 ± 0.81^a^	14.59 ± 0.59^ab^	15.39 ± 0.39^b^	17.99 ± 1.34^c^

*Note:* Values in each row with different letters have significant differences (*p*  < 0.05) (*n =* 9).

### 3.2. Digestive Enzyme Activity

Table 3 shows no significant disparities in the trypsin level among dietary treatments (*p* > 0.05). However, the activities of amylase and lipase in T20 and T30 treatments were higher than those in the T0 and T10 treatment groups (*p* < 0.05).

**Table 3:** Digestive enzyme activity of Yangtze sturgeon fed different dietary starch levels (M ± SE).

Parameters	Dietary starch levels			
	T0	T10	T20	T30
Amylase (U/mg protein)	14.30 ± 0.86^a^	13.70 ± 0.65^a^	15.95 ± 0.91^b^	16.80 ± 0.60^b^
Lipase (U/mg protein)	2.99 ± 0.20^a^	2.94 ± 0.13^a^	3.37 ± 0.21^b^	3.45 ± 0.21^b^
Trypsin (U/mg protein)	8.12 ± 0.67	7.98 ± 0.47	8.58 ± 0.41	8.49 ± 0.34

*Note:* Values in each row with different letters have significant differences (*p*  < 0.05) (*n* = 9).

### 3.3. Muscle Key Metabolic Enzyme Activity

Table 4 indicates the influence of dietary starch levels on muscle glucose key metabolic enzymes. The muscle HK, GK, PFK, PK, and glucose‐6‐phosphate dehydrogenase (G6PDH) activities increased considerably as the starch levels increased (*p* < 0.05). Moreover, the table reveals no considerable differences in the muscle phosphoenolpyruvate carboxykinase (PEPCK) activities in the treatment groups (*p*  > 0.05).

**Table 4:** Muscle key metabolic enzyme activity of Yangtze sturgeon fed different dietary starch levels (M ± SE).

Parameters	Dietary starch levels			
	T0	T10	T20	T30
HK (U/g protein)	7.25 ± 0.38^a^	7.89 ± 0.52^ab^	8.56 ± 0.11^b^	8.45 ± 0.26^b^
GK (IU/g protein)	2.81 ± 0.14^a^	3.04 ± 0.05^ab^	3.18 ± 0.10^b^	3.14 ± 0.20^b^
PK (U/g protein)	383.65 ± 1.99^a^	426.45 ± 39.42^b^	438.60 ± 19.90^b^	435.40 ± 4.23^b^
PFK (U/g protein)	184.27 ± 15.60^a^	206.71 ± 9.24^b^	210.37 ± 5.11^b^	193.40 ± 11.66^ab^
PEPCK (U/g protein)	12.71 ± 1.37	13.75 ± 1.23	13.27 ± 1.07	12.66 ± 1.41
G6PDH (U/g protein)	32.99 ± 1.90^a^	38.05 ± 0.79^b^	37.83 ± 0.38^b^	36.90 ± 0.83^b^

*Note:* Values in each row with different letters have significant differences (*p*  < 0.05) (*n =* 9).

### 3.4. Muscle Antioxidant Indices

Table 5 displays the influence of dietary starch levels on the muscle antioxidant indices. With the increase in starch levels, the muscle SOD, GSH‐PX and CAT activities significantly increased (*p*  < 0.05), and the muscle MDA contents showed the opposite correlation with starch levels (*p*  < 0.05). In addition, under the T30 treatment, the muscle SOD and GSH‐PX presented higher activities than those of other treatments (*p*  < 0.05), and the muscle MDA contents in the T30 treatment were lower (*p*  < 0.05).

**Table 5:** Muscle antioxidant enzyme activity of Yangtze sturgeon fed different dietary starch levels (M ± SE).

Parameters	Dietary starch levels			
	T0	T10	T20	T30
SOD (IU/mg protein)	7.92 ± 0.05^a^	8.42 ± 0.10^a^	9.10 ± 0.25^b^	9.28 ± 0.62^b^
GSH‐PX (U/g protein)	57.65 ± 3.74^a^	62.75 ± 1.47^b^	71.69 ± 2.20^c^	75.98 ± 2.61^c^
CAT (U/mg protein)	1.41 ± 0.09	1.46 ± 0.07	1.55 ± 0.02	1.49 ± 0.07
MDA (umol/g protein)	0.88 ± 0.05	0.85 ± 0.03	0.90 ± 0.04	0.86 ± 0.05

*Note:* Values in each row with different letters have significant differences (*p*  < 0.05) (*n =* 9).

### 3.5. Nutritional Analysis of Dorsal Muscle

To reveal the effects of muscle nutrition of Yangtze sturgeon under different dietary starch levels, the basic nutrient components, amino acids, and fatty acids in the dorsal muscle were focused on. As shown in Table 6, 23 fatty acids were detected in dorsal muscle, including 9 saturated fatty acids (SFAs, 4 monounsaturated fatty acids (MUFAs), and 10 PUFAs. At different levels of dietary starch, there were no significant differences in SFA content between groups, and MUFA and PUFA content decreased with increasing dietary starch content. The proportion of omega‐6 PUFAs and omega‐3 PUFAs (ΣPUFA (*n* – 6)/ΣPUFA(*n* – 3)) increased with dietary starch levels. Nutritional evaluation of fatty acids showed that the values of IA and IT increased with rising dietary starch levels, while PI showed a decreasing trend. Total fatty acid content decreased with increasing dietary starch content.

**Table 6:** Fatty acid composition (g/100 g muscle) and nutritional value evaluation of Yangtze sturgeon fed different dietary starch levels (M ± SE).

Parameters	Dietary starch levels			
	T0	T10	T20	T30
C4:0	–	–	–	–
C6:0	–	–	–	–
C8:0	–	–	–	–
C10:0	–	–	–	–
C11:0	–	–	–	–
C12:0	0.0046 ± 0.01^a^	0.0044 ± 0.01^a^	0.0045 ± 0.01^a^	0.0051 ± 0.01^b^
C13:0	–	–	–	–
C14:0	0.3203 ± 0.01^b^	0.3081 ± 0.01^ab^	0.3135 ± 0.01^ab^	0.3033 ± 0.01^a^
C14:1n5	–	–	–	–
C15:0	0.0478 ± 0.01^a^	0.0473 ± 0.01^a^	0.0476 ± 0.01^a^	0.0449 ± 0.01^b^
C15:1n5	–	–	–	–
C16:0	4.1138 ± 0.05	4.0308 ± 0.06	4.0729 ± 0.02	4.0445 ± 0.01
C16:1n7	0.4853 ± 0.01^c^	0.4577 ± 0.01^b^	0.4594 ± 0.01^b^	0.4319 ± 0.01^a^
C17:0	0.1062 ± 0.01^b^	0.0967 ± 0.01^a^	0.1070 ± 0.01^b^	0.0915 ± 0.01^a^
C17:1n7	–	–	–	–
C18:0	0.9817 ± 0.01	1.0119 ± 0.01	0.9931 ± 0.01	1.0224 ± 0.01
C18:1n9t	–	–	–	–
C18:1n9c	6.4657 ± 0.07^c^	6.4001 ± 0.09^bc^	6.1656 ± 0.03^ab^	6.0771 ± 0.01^a^
C18:2n6t	–	–	–	–
C18:2n6c	6.9264 ± 0.07^b^	6.8489 ± 0.10^b^	6.5222 ± 0.03^a^	6.2991 ± 0.03^a^
C20:0	0.0313 ± 0.01^b^	0.0325 ± 0.01^b^	0.0293 ± 0.01^a^	0.0296 ± 0.01^ab^
C18:3n6 ##	0.2347 ± 0.01^a^	0.2400 ± 0.01^a^	0.2599 ± 0.01^b^	0.2980 ± 0.01^c^
C20:1	0.1843 ± 0.01^ab^	0.1900 ± 0.01^b^	0.1787 ± 0.01^a^	0.1812 ± 0.01^ab^
C18:3n3 #	0.5771 ± 0.01^ab^	0.5780 ± 0.01^ab^	0.5875 ± 0.01^ab^	0.5172 ± 0.01^a^
C21:0	–	–	–	–
C20:2	0.2555 ± 0.01^a^	0.2684 ± 0.01^b^	0.2534 ± 0.01^a^	0.2626 ± 0.01^ab^
C22:0	0.0125 ± 0.01^c^	0.0118 ± 0.01^b^	0.0103 ± 0.01^a^	–
C20:3n6	0.1466 ± 0.01^a^	0.1545 ± 0.01^b^	0.1681 ± 0.01^c^	0.1859 ± 0.01^d^
C22:1n9	0.0243 ± 0.01^b^	0.0223 ± 0.01^ab^	0.0211 ± 0.01^a^	0.0232 ± 0.01^ab^
C20:3n3	0.0448 ± 0.01^a^	0.0493 ± 0.01^b^	0.0448 ± 0.01^a^	0.0434 ± 0.01^a^
C20:4n6 ##	0.2675 ± 0.01^b^	0.2531 ± 0.01^ab^	0.2519 ± 0.01^a^	0.2907 ± 0.01^c^
C23:0	–	0.0191 ± 0.01^a^	0.0186 ± 0.01^a^	0.0262 ± 0.01^b^
C22:2n6	0.0299 ± 0.01^a^	0.0326 ± 0.01^a^	0.0324 ± 0.01^a^	0.0434 ± 0.01^b^
C24:0	–	–	–	–
C20:5n3 #	0.6633 ± 0.01^c^	0.6252 ± 0.01^bc^	0.6170 ± 0.01^b^	0.5694 ± 0.01^a^
C24:1n9	–	–	–	–
C22:6n3 #	1.2774 ± 0.02^b^	1.2768 ± 0.02^b^	1.2722 ± 0.01^b^	1.2064 ± 0.01^a^
ΣSFA	5.6183 ± 0.07	5.5626 ± 0.08	5.5968 ± 0.03	5.5675 ± 0.01
ΣMUFA	7.1597 ± 0.07^c^	7.0701 ± 0.10^bc^	6.8248 ± 0.03^ab^	6.7134 ± 0.01^a^
ΣPUFA	10.168 ± 0.12^b^	10.0585 ± 0.14^b^	9.7561 ± 0.05^ab^	9.4535 ± 0.06^a^
ΣPUFA (*n –* 3)	2.5178 ± 0.04^b^	2.4801 ± 0.03^b^	2.4767 ± 0.01^b^	2.2929 ± 0.03^a^
ΣPUFA (*n* *–* 6)	0.5022 ± 0.01^a^	0.4932 ± 0.01^a^	0.5118 ± 0.01^a^	0.5887 ± 0.01^b^
ΣPUFA (*n* − 6)/ΣPUFA(*n* − 3)	0.1995 ± 0.01^a^	0.1989 ± 0.01^a^	0.2067 ± 0.01^b^	0.2568 ± 0.01^c^
Total fatty acids	23.2012 ± 0.26^b^	22.9595 ± 0.33^ab^	22.4312 ± 0.12^ab^	21.9971 ± 0.08^a^

IA	0.3116 ± 0.01^a^	0.3075 ± 0.01^b^	0.3215 ± 0.01^c^	0.3255 ± 0.01^d^
IT	0.4675 ± 0.01^a^	0.4685 ± 0.01^a^	0.4759 ± 0.01^b^	0.4979 ± 0.01^c^
PI	0.4717 ± 0.01^b^	0.4719 ± 0.01^b^	0.4639 ± 0.01^b^	0.4390 ± 0.01^a^

*Note:* – means not detected; # means PUFA (*n –* 3); ## means PUFA (*n* – 6). Values in each row with different letters have significant differences (*p*  < 0.05).

Amino acid composition is demonstrated in Table 7. The values of ∑EAA/∑TAA and ∑EAA/∑NEAA decreased with increasing levels of dietary starch, while ∑DAA/∑TAA showed a decreasing trend. The nutritional value evaluations of amino acids are demonstrated in Table 8. Valine was the first limiting amino acid at all four dietary starch levels (0%, 10%, 20%, and 30%). FI values for amino acids in the dorsal muscles were highest at T0 (0% dietary starch), decreased significantly at T10 (10% dietary starch) and T30 (30% dietary starch), and were lowest at T20 (20% dietary starch).

**Table 7:** Amino acid composition (g/100 g muscle) of Yangtze sturgeon fed different dietary starch levels (M ± SE).

Parameters	Dietary starch levels			
	T0	T10	T20	T30
Aspartic acid	1.61 ± 0.02	1.56 ± 0.02	1.61 ± 0.04	1.54 ± 0.01
Threonine	0.74 ± 0.01	0.72 ± 0.01	0.74 ± 0.02	0.70 ± 0.01
Serine	0.70 ± 0.01^b^	0.67 ± 0.01^ab^	0.70 ± 0.01^b^	0.65 ± 0.01^a^
Glutamic acid	2.52 ± 0.04	2.51 ± 0.04	2.57 ± 0.07	2.48 ± 0.01
Glycine	0.87 ± 0.02	0.87 ± 0.01	0.87 ± 0.02	0.85 ± 0.01
Alanine	0.92 ± 0.02	0.92 ± 0.01	0.90 ± 0.02	0.89 ± 0.01
Valine	0.73 ± 0.01	0.76 ± 0.01	0.73 ± 0.02	0.75 ± 0.01
Methionine	0.53 ± 0.01	0.52 ± 0.01	0.54 ± 0.01	0.51 ± 0.01
Isoleucine	0.73 ± 0.01	0.75 ± 0.01	0.73 ± 0.02	0.75 ± 0.02
Leucine	1.27 ± 0.02	1.26 ± 0.02	1.27 ± 0.03	1.25 ± 0.01
Tyrosine	0.53 ± 0.01^a^	0.57 ± 0.01^b^	0.58 ± 0.01^b^	0.57 ± 0.01^b^
Phenylalanine	0.61 ± 0.01	0.64 ± 0.01	0.65 ± 0.01	0.63 ± 0.01
Lysine	1.49 ± 0.02	1.49 ± 0.02	1.51 ± 0.04	1.49 ± 0.01
Histidine	0.50 ± 0.01	0.49 ± 0.01	0.48 ± 0.01	0.49 ± 0.01
Arginine	1.01 ± 0.02	1.02 ± 0.01	1.02 ± 0.02	1.00 ± 0.01
Proline	0.59 ± 0.01	0.58 ± 0.01	0.59 ± 0.01	0.56 ± 0.01
∑TAA	15.35 ± 0.24	15.35 ± 0.22	15.48 ± 0.38	15.13 ± 0.08
∑EAA	6.09 ± 0.09	6.15 ± 0.09	6.16 ± 0.16	6.10 ± 0.05
∑SEAA	1.51 ± 0.026	1.51 ± 0.02	1.50 ± 0.03	1.49 ± 0.01
∑NEAA	7.75 ± 0.12	7.69 ± 0.11	7.81 ± 0.19	7.55 ± 0.02
∑DAA	5.92 ± 0.09	5.86 ± 0.08	5.95 ± 0.15	5.77 ± 0.02
∑EAA/∑TAA/%	39.69 ± 0.02^a^	40.07 ± 0.05^bc^	39.84 ± 0.04^ab^	40.29 ± 0.12^c^
∑EAA/∑NEAA/%	78.64 ± 0.03^a^	79.98 ± 0.23^bc^	78.91 ± 0.09^ab^	80.79 ± 0.49^c^
∑DAA/∑TAA/%	38.58 ± 0.01^c^	38.18 ± 0.07^ab^	38.43 ± 0.02^bc^	38.12 ± 0.08^a^

*Note:* Values in each row with different letters have significant differences (*p*  < 0.05).

**Table 8:** Amino acid nutritional value evaluation of Yangtze sturgeon fed different dietary starch levels (M ± SE).

Parameters	Dietary starch levels			
	T0	T10	T20	T30
Isoleucine	104.75 ± 0.51^a^	113.30 ± 0.39^ab^	112.08 ± 2.29^ab^	117.12 ± 3.04^b^
Leucine	104.52 ± 0.56^a^	108.48 ± 0.26^ab^	111.17 ± 2.05^b^	111.27 ± 1.25^b^
Lysine	155.70 ± 1.02^a^	163. 26 ± 0.40^ab^	168.33 ± 3.81^b^	168.55 ± 1.98^b^
Phenylalanine + Tyrosine	109.01 ± 0.99^a^	121.29 ± 0.39^b^	125.40 ± 1.99^b^	124.31 ± 1.74^b^
Threonine	106.53 ± 0.06^a^	108.16 ± 0.53^ab^	112.82 ± 2.04^b^	109.39 ± 0.38^ab^
Valine	83.52 ± 0.77^a^ ^∗^	90.94 ± 0.54^b^ ^∗^	89.67 ± 1.93^ab^ ^∗^	93.60 ± 2.25^b^ ^∗^
FI	2.40 ± 0.01^c^	2.29 ± 0.01^b^	2.23 ± 0.01^a^	2.30 ± 0.01^b^

Note:  ^∗^ means the first limiting amino acid. Values in each row with different letters have significant differences (*p*  < 0.05).

## 4. Discussion

### 4.1. Effects of Dietary Starch Levels on Digestive Enzyme Activity of Juvenile Yangtze Sturgeon

Digestive enzymes break down food into small particles that can be absorbed, which is a key step in nutrient absorption [20]. In previous studies, the activities of digestive enzymes directly determined the digestibility of dietary carbohydrate for fish [21]. In our work, lipase and amylase activities increased greatly with increasing starch levels, which implies that Yangtze sturgeon can tolerate ≤30% dietary starch levels via the regulation of digestive enzyme activities. A similar finding was observed in *R. canadum* [22] and *Ocyurus chrysurus* [23]; however, in *Monopterus albus*, the increase in starch levels from 16% to 40% greatly suppressed amylase activities [3]. The different rustults could potentially be attributed to variations in the digestive and absorptive capabilities of fish concerning carbohydrates. An overabundance of dietary starch might lead to intestinal dysfunction, a condition that arises from an imbalance in the intestinal microbiota and a subsequent decrease in the production of short‐chain fatty acids [12].

### 4.2. Effects of Dietary Starch Levels on Key Metabolic Enzyme Activity of Juvenile Yangtze Sturgeon

The glycolysis is limited by a series of key metabolic enzymes and may result from the presence of certain dietary starch levels in some species, including *Sparus aurata* [24], *M. salmoides* [25], *Epinephelus coioides* [26], and *O. chrysurus* [23]. HK catalyzes the first step of cellular glucose catabolism for energy production, whereas GK shunts excess glucose toward lipid and glycogen synthesis. PK catalyzes the final step of glycolysis and governs the rates of pyruvate and ATP generation. In this research, dietary starch induced considerable increases in the levels of glycolytic enzymes (HK, GK, PFK, and PK). These data indicate that glycolytic enzyme (HK, GK, PFK, and PK) activity is adaptable to dietary carbohydrates in both fish species, suggesting that PK mediates postprandial glucose utilization in Yangtze sturgeon. *M. salmoides* [25] and *E. coioides* [26] showed similar findings.

In this study, the G6PDH activity was improved as Yangtze sturgeon were fed with an increased starch diet, which is consistent with those in previous study [27, 28]. These data indicates that G6PDH activity is adaptable to dietary carbohydrates, implying that this enzyme mediates postprandial glucose utilization in Yangtze sturgeon. G6PDH contribute to the generation of NADPH, which is essential for fatty acid synthesis [29]. In the present study, higher G6PDH activity coincided with greater whole‐body lipid content in *C. undecimalis* fed an increased‐starch diet.

### 4.3. Effects of Dietary Starch Levels on Antioxidant Capability of Juvenile Yangtze Sturgeon

The findings reveal the substantial increase in the antioxidant enzymes and increase the activities of antioxidant enzymes. Redox homeostasis is very important for fish immunity and may be subject to various factors involving feed composition, such as carbohydrates and lipid [30, 31]. The findings reveal the substantial increase in the activities of antioxidant enzymes, including CAT, SOD, and GSH‐PX, in the muscle and liver of Yangtze sturgeon with the increase in dietary starch levels, which accorded with the findings on *Mylopharyngodon piceus* [11] and *Megalobrama amblycephala* [30]. A previous study confirmed that an increased antioxidant capability improves the metabolic homeostasis and health status of fish [32, 33]. The above findings on antioxidant enzymes suggest that starch as an alternative energy source may contribute to the oxidative protection of Yangtze sturgeon, as proven by a low concentration of hepatic MDA in a starch‐rich diet. On the one hand, Sagone et al. [34] demonstrated that glucose could directly scavenge OH^-^ radicals. On the other hand, dietary carbohydrates have been reported to activate the pentose phosphate pathway [35, 36]. This pathway generates NADPH as reducing power, which in turn promotes CAT and GPX activities.

### 4.4. Effects of Dietary Starch Levels on Muscle Nutrient Composition of Juvenile Yangtze Sturgeon

In fish, the muscle serves as the primary site for nutrient deposition [37]. The results demonstrate that the ratios of ΣPUFA (*n* – 6)/ΣPUFA(*n* – 3), IA, IT, ∑EAA/∑TAA, and ∑EAA/∑NEAA all increased with rising starch content in the diet. Conversely, ∑DAA/∑TAA and PI showed a decreasing trend with higher starch levels. The elevated ∑EAA/∑TAA and ∑EAA/∑NEAA ratios indicate that increased dietary starch enhances the proportion of essential amino acids in fish tissues. This suggests that higher starch content may promote protein synthesis and improve nitrogen utilization efficiency. With increasing starch content, the crude fat content in dorsal muscle rises, leading to fat accumulation and obesity in fish. The levels of MUFAs and PUFAs decrease proportionally with starch content, while the IA and IT values increase due to the reduction of these unsaturated fatty acids. This alteration not only compromises the antioxidant capacity of fish but also deteriorates the fatty acid quality in dorsal muscle [38].

Starch, as a primary carbohydrate source in fish feed, significantly influences growth performance and energy metabolism. Studies have demonstrated that increasing dietary starch levels elevate body fat content in wuchang bream (*Megalobrama amblycephala*) [39], silver crucian carp (*Carassius auratus gibelio*) [40], and crucian carp (*Carassius auratus*) [41]. Transcriptomic analysis revealed that excessive dietary starch intake downregulates PPARα expression, a master regulator of lipid metabolism, in Largemouth bass (*Micropterus salmoides*) [25]. Consistent with previous findings, our study observed that higher starch content increased crude fat content and hepatosomatic index (HSI) in Yangtze sturgeon. Fish primarily derive energy from proteins, fats, and carbohydrates (e.g., starch). The protein‐sparing effect, whereby increased dietary starch availability decreases amino acid utilization for energy metabolism, is particularly pronounced in starch‐utilizing omnivorous species such as tilapia and common carp [42–45]. Although excessive starch promotes adiposity, moderate starch supplementation optimizes protein utilization efficiency, potentially improving amino acid retention [46]. Studies on crucian carp have shown that increasing dietary starch levels reduces crude protein content in muscle tissue, consistent with our findings. However, appropriate dietary starch supplementation in crucian carp enhances protein deposition efficiency in muscle, suggesting that starch inclusion improves protein utilization in feed and consequently reduces aquaculture production costs [7] Notably, Yangtze sturgeon is an omnivorous species, and our results indicate that ∑EAA/∑TAA and ∑EAA/∑NEAA ratios increased with starch content, further supporting the hypothesis that optimal starch supplementation enhances amino acid sparing, directing more amino acids toward growth rather than catabolism.

## 5. Conclusion

These findings demonstrated that 20%–30% starch could enhance metabolic and immunity performance in juvenile Yangtze sturgeon, which provided valuable insights for the aquaculture industry. This not only benefits the economic viability of aquaculture operations but also contributes to the conservation and sustainable development of the Yangtze sturgeon population. However, the relation between the health status of juvenile Yangtze sturgeon and dietary starch levels should be given more attention to clarify the appropriate level of dietary starch. Future research could further explore the long‐term effects of this starch‐supplemented diet on the reproductive performance of Yangtze sturgeon, as well as optimize the feed formula based on different growth stages of the fish.

## Author Contributions

Bin He, Bo Zhou, and Tao Yan planned the sample collection. Data analysis, preliminary drafting, and approval of the final manuscript were conducted by Tao Yan, Xu Kuang, Qingzhi Li, Tingting Wu, and Yunyi Xie.

## Funding

This research was funded by the Innovation Ability Improvement Special Youth Foundation of Sichuan (Grant 2019QNJJ‐013) and Special Project of Fishery Resources and Environment Investigation in Key Waters of Southwest China (Grant CJW2023034).

## Ethics Statement

The current study followed a standard working methodology approved by the Institutional Animal Care and Use Committee (IACUC) of Fisheries Research Institute, Sichuan Academy of Agricultural Sciences (Approval Number 20230608002A).

## Conflicts of Interest

The authors declare no conflicts of interest.

## Data Availability

All data shall be provided upon request.
